# Shared Adversity Increases Team Creativity Through Fostering Supportive Interaction

**DOI:** 10.3389/fpsyg.2018.02309

**Published:** 2018-11-23

**Authors:** Brock Bastian, Jolanda Jetten, Hannibal A. Thai, Niklas K. Steffens

**Affiliations:** ^1^Melbourne School of Psychological Sciences, The University of Melbourne, Melbourne, VIC, Australia; ^2^School of Psychology, University of Queensland, St Lucia, QLD, Australia

**Keywords:** pain, adversity, creativity, group interaction, social support, team climate, cooperation, innovation

## Abstract

In the current era, building more innovative teams is key to organizational success, yet there is little consensus on how best to achieve this. Common wisdom suggests that positive reinforcement through shared positive rewards builds social support within teams, and in turn facilitates innovation. Research on basic group processes, cultural rituals, and the evolution of pro-group behavior has, however, revealed that sharing adverse experiences is an alternative path to promoting group bonding. Here, we examined whether sharing an adverse experience not only builds social support within teams, but also in turn enhances creativity within novel teams. Drawing on behavioral observation of an experimental group interaction we find evidence that sharing an adverse (vs. non-adverse) experience leads to increased supportive interactions between team members and this in turn boosts creativity within a novel team. These effects were robust across different indicators of creativity: objective measures of creativity, third party ratings of the creativity of group products, and participants' own perceptions of group creativity. Our findings offer a new perspective from which to understand how best to boost innovation and creative output within teams.

## Introduction

Boosting creativity and innovation within teams is not only critical for group and organizational success, but is also a key solution to many modern challenges facing world economies (Paulus and Yang, 2000; Haslam et al., 2013). In unpacking sources of creative thinking and innovation, research has focused on cognitive and motivational processes within groups (Scott and Bruce, 1994; Unsworth, 2001; Brown and Paulus, 2002; Paulus and Brown, 2003; Nijstad and Stroebe, 2006; De Dreu et al., 2011) as well as leadership and group dynamics within teams (Redmond et al., 1993; Perry-Smith and Shalley, 2003; Mumford and Licuanan, 2004; Perry-Smith, 2006; Hülsheger et al., 2009). One factor identified as critical to the emergence of creative idea generation within teams is a supportive team environment: Interactions that produce a supportive group environment, thereby lowering the fear of disagreement or ridicule, have been found to facilitate the generation of novel and divergent input into group products (Edmondson, 1999; Baer and Frese, 2003; Edmondson and Mogelof, 2006; Mathisen et al., 2008; Carbonell and Rodríguez-Escudero, 2009; Hülsheger et al., 2009; Pirola-Merlo, 2010).

What is less clear, however, is how to foster a supportive team environment. The emergence of consultant-led team building exercises have aimed to address this issue using tools such as positive reinforcement, goal setting, and problem solving (Klein et al., 2009). Comfortable workplace environments, popularized by Silicon Valley start-ups (Blair, 2017) complete with break-out areas, games, bean bags, and bright colors, have also been employed to similar ends. These approaches are consistent with research showing that shared positive feelings—like happiness and excitement—promote social bonding and integration (Spoor and Kelly, 2004; Fischer and Manstead, 2008; Niedenthal and Brauer, 2012) and in turn have been linked to productivity (although not creativity or innovation specifically; Knight and Eisenkraft, 2015). Yet, research focusing on basic group processes, cultural rituals, and evolved mechanisms underlying pro-group behavior suggests an alternative: sharing negative or adverse experiences is especially effective in promoting group bonding and commitment (Harper, 2006; Henrich, 2009; Bastian et al., 2014a; Whitehouse et al., 2014, 2017). In the current research, we focus on ways other than evoking positive emotions to boost creativity. In particular, we focused on the role of shared adversity, and examined whether this experience fosters the kind of supportive team environment that promotes creativity and innovation within teams.

A better understanding of this possibility is important for two reasons. First, if true it would provide a previously unexamined perspective on how social supprt and, in turn, creativity can be fosterd within teams. Teams are often confronted with adverse and challenging circumstances, and providing insight into how and why such circumstances may hold the potential for boosting creatvity would throw new light on not only how teams might respond to these events, but also why such events may be valuable and important for the emergence of valued group processes. Second, it would extend previous work showing that shared adversity is a powerful trigger for group bonding by examining the possibility that these experinces may also lead to other positive group outcomes.

Below we first examine the evidence for the link between supportive social interactions and creativity. Next, we review evidence that shared adversity is an especially powerfully trigger for fostering supportive team interactions, which should therefore provide an avenue through which shared adversity may foster creativity in teams.

### Supportive team social interaction and creativity

Innovation with teams is often driven by the somewhat random process of developing unusual combinations from divergent perspectives and seeking feedback on ideas that push boundaries, but which also pose the risk of making team members look ignorant or even incompetent by others (Simonton, 1999). To achieve this, people need to feel comfortable that they can express divergent and often risky ideas without fear of ridicule. The aspect of a team environment which allows team members to relax their guard and engage openly in the exploration of risky ideas has been referred to as psychological safety—the shared belief held by members of a team that others will respond positively when one exposes one's thoughts, such as by asking a question, seeking feedback, reporting a mistake, or proposing a new idea (West, 1990, 2002; Edmondson, 1999, 2002; Baer and Frese, 2003; Edmondson and Mogelof, 2006). Consistent with this, research has found that sensitivity to the potential negative reactions of others (e.g., disagreement, ignoring, or ridicule) is a key factor that inhibits the creative process (Camacho and Paulus, 1995; Paulus and Korde, 2013) and teams lacking in psychological safety are less likely to engage in the behavioral hallmarks of creativity (West, 1990).

Paulus and Brown (2007) have argued that fostering a team climate that allows people to feel comfortable expressing divergent ideas requires the emergence of “some type of “bridge” that binds the group members together” (p. 256). Sharing experiences with others may be an important avenue through which to build this type of bridge between team members.

There has been limited work examining the link between shared experiences and creativity. Consistent with the recent trend toward building comfortable and pleasant shared office spaces, research has linked the experience of positive affect to creativity due to its capacity to increase cognitive flexibility (Baas et al., 2008; De Dreu et al., 2008). Yet, this work has focused on individuals, and whether sharing positive affective experiences with others boosts the creative output of teams is unclear. Rather than addressing this question, in the current work we move away from the focus on positive experiences and explore whether creativity can be boosted in alternative ways. Specifically, we focus on whether sharing an adverse experience might lead to the kind of supportive team social interactions that encourage the creative process. We see value in this research question for two reasons. The first is to link work in the field of cultural anthropology to research applicable to organizations and team-based processes. There is now a large body of work (see below) which aims to understand why cultural practices emerged and persisted within various societies. Central to this endeavor is understanding factors the promote cohesive, cooperative, and functioning societies, all of which are similarly of interest to groups and organizations aiming to foster similar group processes. The second is to provide a different perspective on shared experiences and how they impact on group functioning. As noted above, there has been an assumption that positive experiences are a primary route through which positive group processes emerge. While this is no doubt true, teams commonly also experience adversity, and understanding that there may be additional value in these negative and adverse experiences provides a framework from which to better respond to, and build from, these types of interactions and associated outcomes.

### Shared adversity and supportive team social interaction

Sharing an experience with others has been shown to be a powerful predictor of interpersonal attraction (more so than similarity; Pinel et al., 2006) and increases cooperative behavior within groups (Wiltermuth and Heath, 2009). Certain factors within a shared experience may be especially potent in bonding group members together. For instance, synchronous, complementary movement has been found to promote interpersonal and group bonding more so compared to asynchronous, non-complementary movement (Wiltermuth and Heath, 2009; Paladino et al., 2010; Koudenburg et al., 2015). This provides one reason why synchronous and complementary movement is often employed within military marching and ritual ceremonies.

Beyond synchrony, sharing powerful affective experiences may also forge social bonds. Shared positive experiences have been shown to serve a broad affiliative function, enabling bonding and the creation of social relationships across group boundaries (Spoor and Kelly, 2004; van der Schalk et al., 2011). In contrast, shared negative experiences appear to serve a boundary demarcation function, alerting group members to possible threats and emphasizing distinctions between in-group and out-group members. In this way, shared experiences may promote an exclusive focus on the in-group and foster selective social integration and support (especially when these experiences are caused by factors external to the group; see Knight and Eisenkraft, 2015). In contrast to more pleasant or positive shared experiences, experiences that involve challenging or adverse conditions may promote greater interdependency between in-group members (Whitehouse et al., 2017).

This function of such experiences is buttressed by the observation that social rituals frequently elicit unpleasant or painful shared experiences (Whitehouse, 1996). Indeed, Durkheim (1912/1995) argued that painful experiences function to promote solidarity within groups and evidence for this assertion is borne out by observations of group behavior in response to large scale disasters (Penner et al., 2005; Gelfand et al., 2011; Harrington and Gelfand, 2014; Vezzali et al., 2016) or the trauma of war (Whitehouse et al., 2017). Experimental research also supports this effect showing that when people endure painful group rituals, or are induced to experience fear, stress, or pain within a controled laboratory setting, they are more likely to behave in trusting and cooperative ways (Schachter, 1959; Gump and Kulik, 1997; von Dawans et al., 2012; Xygalatas et al., 2013; Bastian et al., 2014a).

We argue that sharing adverse experiences may be especially likely to promote supportive interactions within groups and this is critical to creative idea generation. This is because such experiences enhance commitment to the group (Whitehouse et al., 2017), leading individuals to mutually seek and provide support to one another (e.g., Molero et al., 2011; Haslam et al., 2018). Taylor et al. (2000, 2006) have referred to this as the tend-and-befriend motivation, suggesting that affiliating with others is a common and basic response to pain and stress. This is consistent with work showing that existential threats increase interest in pictures of people more so than in pictures of objects (Zhou et al., 2009) and that fear of electric shock motivates physical proximity (Shaver and Klinnert, 1982; Rofé, 1984). Other work shows that social pain increases sensitivity to social information (Gardner et al., 2000; Pickett et al., 2004) and the perceived value of relationships (Maner et al., 2007), as well as promotes non-conscious behaviors which enhance social relations, such as increased mimicry (Lakin and Chartrand, 2003) and affiliative social tuning (Sinclair et al., 2005).

The evidence suggests that when people are exposed to stress, rejection, fear, or pain, they seek affiliation with, and to provide support to, others within their social environment (see also Haslam et al., 2018). Moreover, these experiences elicit interdependency and commitment to the group, due to their tendency to motivate affiliation and solidarity, encourage cooperation and trust, and increase reliance on the group as a source of support. It is through these various avenues that shared adversity has the potential to foster an environment that is characterized by reduced fear of negative evaluation. This, in turn, should enhance the willingness of individuals to express divergent, risky, and novel ideas, providing a fertile environment for creative idea generation to emerge.

In the current study, we drew on the experience of physical pain as an example of an adverse experience. This is in-line with our other work on the nature and downstream effects of physical pain (Bastian et al., 2011, 2013, 2014b) which we have also extended both empirically and theoretically to a broader range of adverse and unpleasant experiences (Bastian et al., 2014c; Harmon-Jones et al., 2017; Slepian and Bastian, 2017; Bastian, 2018). To this end, we not only examined whether people reported pain in response to our manipulation, but also a broad range of negative responses. Our aim was to examine whether this type of experience fostered supportive team social interactions. Drawing on methods developed and validated in our own previous research, we induced pain through the consumption of hot chilies and wall squats and measured supportive interactions through observation of affiliative behavior such as the extent of eye contact, talking, helping, and encouragement and whether team members appeared to feel comfortable and capable of making a contribution. We measured creativity using objective measures, third party ratings of a team product, as well as team members' self-perceived creativity. In line with our theoretical approach detailed above, we predicted that sharing adversity in the form of a painful (vs. non-painful) experience would foster supportive team social interactions and that this in turn would facilitate increased levels of creativity within each team.

## Methods

This study was carried out in accordance with the recommendations of National Statement on Ethical Conduct in Human Research. The protocol was approved by the Behavioral and Social Sciences Ethical Review Committee, The University of Queensland (approval # 2008001775). All subjects gave written informed consent in accordance with the Declaration of Helsinki. Our use of experimentally induced physical pain is in line with a very large body of research examining responses to physically painful stimuli (e.g., Price et al., 1983). Furthermore, all participants were free to cease engagement with the painful tasks at any time (there was no enforced time limit) or could withdraw from the study at any stage without consequence.

In line with Bastian et al. (2014a) who found medium to large effect sizes, we estimated that a sample size of 190 individuals in at least 52 groups (with 3 to 5 members) would allow for at least 80% power to detect an effect size of *d* = 0.80. We stopped data collection when this sample size was achieved. One hundred and eighty-nine university students (130 female, 59 male; *M*_age_ = 19.64 years)^1^ participated in the study for course credit. Groups of participants were randomly allocated to either a pain condition (group *n* = 27, individual *n* = 94) or a no-pain condition (group *n* = 28, individual *n* = 95). Group sizes ranged between 3 and 5 participants (*n* = 32 had three members, *n* = 22 had four members, *n* = 1 had five members)^2^, with a median of 3 members (*M* = 3.51; *SD* = 0.55). All sessions were videotaped.

### Procedure and measures

The team of participants completed two group-based tasks that served as our experimental manipulation to induce pain (i.e., a consumer preference task and a physical acuity task). Both tasks have been used in our previous research (Bastian et al., 2014a) and have been shown to reliably elicit the experience of both pain and unpleasantness. In these studies, we report all measures, manipulations, and exclusions.

#### Consumer preferences task

Each team of participants were seated around a large table and were told they would be tasting different kinds of food as part of a consumer preference survey. Participants in the pain condition were given a plate of raw (very spicy) Birds Eye chilies which was placed in the center of the table and were instructed to eat as many as possible (yogurt and water were provided). Participants in the no-pain condition were provided with a plate of hard-boiled sweets and were instructed to take one sweet, and when consuming it hold the sweet in their mouth rather than to chew it. Both tasks lasted for 2 min.

#### Physical acuity task

The premise of the second task was to measure participants' physical acuity. This task took place just to the side of the table where the participants had completed the consumer task (above) and they engaged in this task simultaneously and in full view of each other. Participants in the pain condition were asked to perform an upright wall squat, with their back kept straight and their knees bent at 90°, for as long as possible (up to a maximum of 2 min; *M* = 92.18; *SD* = 26.33). Participants in the no-pain condition were asked to balance on one leg for the duration of 2 min and instructed to switch legs and use balance aids as necessary to make sure this was experienced as comfortable.

#### Creativity tasks

We employed two creativity tasks. In the first task participants completed the brick-variant of the Multiple Uses Task (after Guilford, 1967; Tadmor et al., 2012), which involves generating as many ideas as possible for ways in which one can use a brick. Participants were told to perform the task as a team, and to verbalize any ideas as they come up so that they can be recorded on tape. Consistent with Tadmor et al. (2012) teams had 2 min to complete this task.

Two independent coders rated participants' responses in terms of *fluency, flexibility*, and *originality* in idea generation. *Fluency* was captured by the total number of ideas that a group generated. To yield *flexibility*, independent coders employed the coding scheme developed by Markman et al. 2007; (see also Tadmor et al., 2012) coding the number of different semantic categories that the generated ideas fell into (out of a total of 18 categories). Example semantic categories for uses of a brick include Height (e.g., use brick as a stair), Weight (e.g., paperweight), or Active Tool (e.g., hammer). There was substantial overall agreement between the raters' coding of flexibility (Cohen's κ = 0.671, *p* < 0.001).

Finally, we used a point-coding scheme to assess idea *originality* (Runco et al., 1987; Tadmor et al., 2012). Two coders assigned points to each generated idea based on their uniqueness (i.e., very frequent, frequent, infrequent, and very infrequent). Uses falling into very frequent semantic categories (58.81% of all ideas) were assigned one point. Where an example within the category was significantly unique (if mentioned < 5 times) it was awarded two points, or three points (if mentioned < 1 time). Uses falling into frequent semantic categories were awarded two points (29.13% of all ideas), with infrequent examples awarded three points (if mentioned no more than 3 times). Uses belonging to infrequent sematic categories were awarded three points (9.62% of all ideas), with infrequent examples awarded four points (if mentioned no more than once). Finally, ideas falling into the very infrequent semantic categories were awarded four points (2.44% of all ideas). The two coders resolved any discrepancies through discussion.

In the second task, participants worked together as a team to create a work of art. Magazines and miscellaneous stationery were provided along with a piece of A3 paper. Participants were given 2 min of discussion after which they were given 10 min of working time where no verbal communication was allowed to increase the difficulty of the task. Two groups of twenty coders, who were blind to condition, rated the creativity of the artworks. Each group rated half of the 55 artworks. Using a 7-points scale (1 = *not at all*; 7 = *very much*) raters coded each artwork on creativity (9-items: *original, creative, imaginative, artistic, ingenious, innovative, unique, special, distinct*; *M* = 3.63, *SD* = 0.58, α = 0.98), blandness (*dull, exciting, [reversed], bland, fun [reversed], plain, tasteless, boring*; *M* = 3.67, *SD* = 0.54, α = 0.96), and richness (*depth, detail, texture, variety, richness, breadth, coherent, well-composed, integrated*; *M* = 3.57, *SD* = 0.50, α = 0.97).

#### Self-perceived creativity

At the end of the experiment using a 7-points scale (1 = *strongly disagree*; 7 = *strongly agree*) participants indicated their perceived creativity in the idea generation task (“The generated ideas are creative”; “We came up with uses for a brick that are unusual”; “The uses we came up with are original”; *M* = 4.95, *SD* = 1.26, α = 0.79) and the poster task (“ This piece of art is creative”; “We came up with a piece of art that is unusual”; “The piece of art we created is original”; *M* = 5.24, *SD* = 1.38, α = 0.79)^3^.

#### Task ratings

The very last survey involved participants rating the experimental manipulation tasks (painful vs. non-painful) on a range of indicators. To capture their experience of physical pain we used an established rating method that captured two dimensions of a painful experience—intensity and unpleasantness (Price et al., 1983; “Please circle a number to indicate how intense the pain you experienced was”; 1 = *not painful*, 10 = i*ntensely painful*, and “Please circle a number to indicate how unpleasant the pain you experienced was”; 1 = *not bad at all*, 10 = *the most intense bad feeling imaginable*)^4^.

Participants also rated their perceptions of the task using the Appraisal of Life Events Scale (ALES; Ferguson et al., 1999) which is designed to capture how threatening (6-items: *threatening, fearful, worrying, hostile, frightening, terrifying*, α = 0.80) and how challenging (6-items: *enjoyable, challenging, stimulating, exhilarating, informative, exciting*, α = 0.68) both tasks were on a scale from 1 = *very slightly or not at all*, to 5 = *extremely*^5^.

Finally, participants also rated the emotions they experienced during the physical tasks using the discrete emotion scale (DES: Harmon-Jones et al., 2016). This includes subscales for anger (*anger, made, pissed off, rage*, α = 0.86), disgust (*sickened, grossed out, nausea, revulsion*, α = 0.76), fear (*fear, panic, scared, terror*, α = 0.71), anxiety (*worry, dread, nervous, anxiety*, α = 0.82), sadness (*empty, grief, sad, lonely*, α = 0.53), desire (*craving, desire, longing, wanting*, α = 0.75), relaxed (*calm, chilled-out, easygoing, relaxation*, α = 0.86), and positivity (*enjoyment, happy, liking, satisfaction*, α = 0.87) rated on a scale from 1 = *not at all*, to 7 = *an extreme amount*^6^.

#### Supportive interaction ratings

Two independent coders who were blind to the hypotheses of the study coded participants' behavioral interactions across each of the pain manipulation and creativity tasks from video recordings of each session (see Supplementary Materials for coding instructions). Coding of the creativity tasks was conducted first to ensure that the raters were blind to condition where possible. Coding of the pain manipulation tasks (consumer preference and leg squat) were coded as one. Interaction was rated by focusing on the extent of *eye contact, talking* and how much of a *contribution* each participant displayed, as well as overall ratings of how *cooperative* the team was. Supportiveness was rated by focusing on the extent of *helping, encouragement* and the level of *comfort* each participant displayed as well as overall ratings of how *cohesive* the team was. All ratings were made on a scale ranging from 1 (*not at all*) to 5 (*very much*). To assess the reliability of the rating scale, a second coder also coded 60% of all sessions. Overall, the mean intercorrelation between the composite scores based on the ratings of the two coders was *r* = 0.74, indicating a reliable coding. Principle component analysis for ratings of interaction and supportiveness on each of the tasks separately indicated a single factor explaining more than 57% of the variance. We therefore collapsed all scores to form a measure of supportive interaction (*M* = 2.64, *SD* = 0.52, α = 0.92).

The design of our study meant that not only were some of our ratings provided at the team-level (e.g., team supportive interactions, poster ratings), but also the individual data were non-independent (e.g., number of brick uses generated, individual interactions). We therefore analyzed the data examining our focal hypothesis at the team level.

## Results

### Manipulation check

Manipulation checks revealed that participants in the pain condition reported higher pain intensity (*M* = 4.79, *SD* = 2.09) and higher pain unpleasantness (*M* = 4.90, *SD* = 2.23) than those in the no-pain condition [intensity: *M* = 1.50, *SD* = 1.14, *t*_(187)_ = 13.44, *p* < 0.001; unpleasantness: *M* = 1.48, *SD* = 1.17, *t*_(187)_ = 13.20, *p* < 0.001]. This indicated that our pain manipulation was successful.

We also examined responses to the Discrete Emotion Questionnaire and therefore whether our manipulation elicited a broader range of negative and unpleasant emotions. This revealed that participants in the pain condition reported more disgust (*M* = 1.53, *SD* = 0.76), fear (*M* = 1.50, *SD* = 0.74), and anxiety (*M* = 2.18, *SD* = 1.11) compared to those in the no-pain condition [disgust: *M* = 1.06, *SD* = 0.18, *t*_(187)_ = 5.99, *p* < 0.001; fear: *M* = 1.15, *SD* = 0.25, *t*_(187)_ = 4.43, *p* < 0.001; anxiety: *M* = 1.71, *SD* = 0.76, *t*_(187)_ = 3.34, *p* = 0.001]. Participants in the pain condition also experienced less relaxation (*M* = 3.99, *SD* = 1.39) and less positivity (*M* = 3.67, *SD* = 1.29) compared to those in the no-pain condition [relaxation: *M* = 4.59, *SD* = 1.27, *t*_(187)_ = 3.08, *p* = 0.002; positivity: *M* = 4.09, *SD* = 1.32, *t*_(187)_ = 2.23, *p* = 0.027].

Finally, we examined responses to the Appraisal of Life Events Scale. This revealed that participants in the pain condition reported the tasks were more threatening (*M* = 1.53, *SD* = 0.69) and more challenging (*M* = 2.51, *SD* = 0.71) compared participants in the no-pain condition [threatening: *M* = 1.12, *SD* = 0.43, *t*_(187)_ = 4.84, *p* < 0.001; challenging: *M* = 2.08, *SD* = 0.60, *t*_(187)_ = 4.43, *p* < 0.001].

Overall, our findings revealed that the pain condition not only increased the experience of pain (both pain intensity and pain unpleasantness) but more broadly led to increased negative and reduced positive affect. Furthermore, the pain condition was experienced as more threatening and more challenging. This aligns with our broader conceptualization of pain as an intense adverse experience.

### The impact of shared pain on team supportive interaction

We first examined whether the experience of sharing physical pain was associated with increased supportive interactions between team members. To this end we focused on aggregated ratings of each team across all team tasks in the study (i.e., the pain induction, the brick task, the poster preparation phase, and the poster creation phase). As anticipated, this revealed that teams that shared the painful tasks were rated as exhibiting more supportive interaction (*M* = 2.85, *SD* = 0.54) compared to teams that shared the control tasks [*M* = 2.45, *SD* = 0.41; *t*_(55)_ = 3.15, *p* = 0.003, *d* = 0.83, 95% CI(−0.659, −0.141)]. Inspection of the association between (continuously measured) pain ratings and team supportive interaction revealed a similar pattern, with pain intensity (*r* = 0.391, *p* = 0.003) and pain unpleasantness (*r* = 0.415, *p* = 0.002) both significantly correlated with team supportive interaction. Also, in line with past research, and as predicted, supportive interactions were significantly and positively correlated with all measures of creativity (see Table 1). Furthermore, providing evidence for the convergent validity of our creativity measures, many of these divergent indices of creativity were also related to each other.

**Table 1:** Correlations between measures of creativity and supportive interaction.

	**1**	**2**	**3**	**4**	**5**	**6**	**7**	**8**
1. Supportive interaction							
2. Idea generation: Fluency	0.50^***^						
3. Idea generation: Flexibility	0.43^***^	0.82^***^					
4. Idea generation: Originality	0.38^**^	0.89^***^	0.83^***^				
5. Poster creativity	0.34^*^	0.36^**^	0.29^*^	0.22			
6. Poster richness	0.25	0.22	0.14	0.08	0.86^***^		
7. Poster blandness	−0.26	−0.19	−0.16	−0.04	−0.81^***^	−0.85^***^	
8. Self-rated brick creativity	0.42^***^	0.46^***^	0.49^***^	0.40^***^	0.41^***^	0.25	–0.30^*^
9. Self-rated poster creativity	0.39^**^	0.26	0.20	0.24	0.24^*^	0.07	–0.10	0.49^***^

* *p < 0.05*,

** *p < 0.01*,

*** *p < 0.001*.

### The indirect effect of shared pain on creativity through team supportive interaction

**Figure 1:**
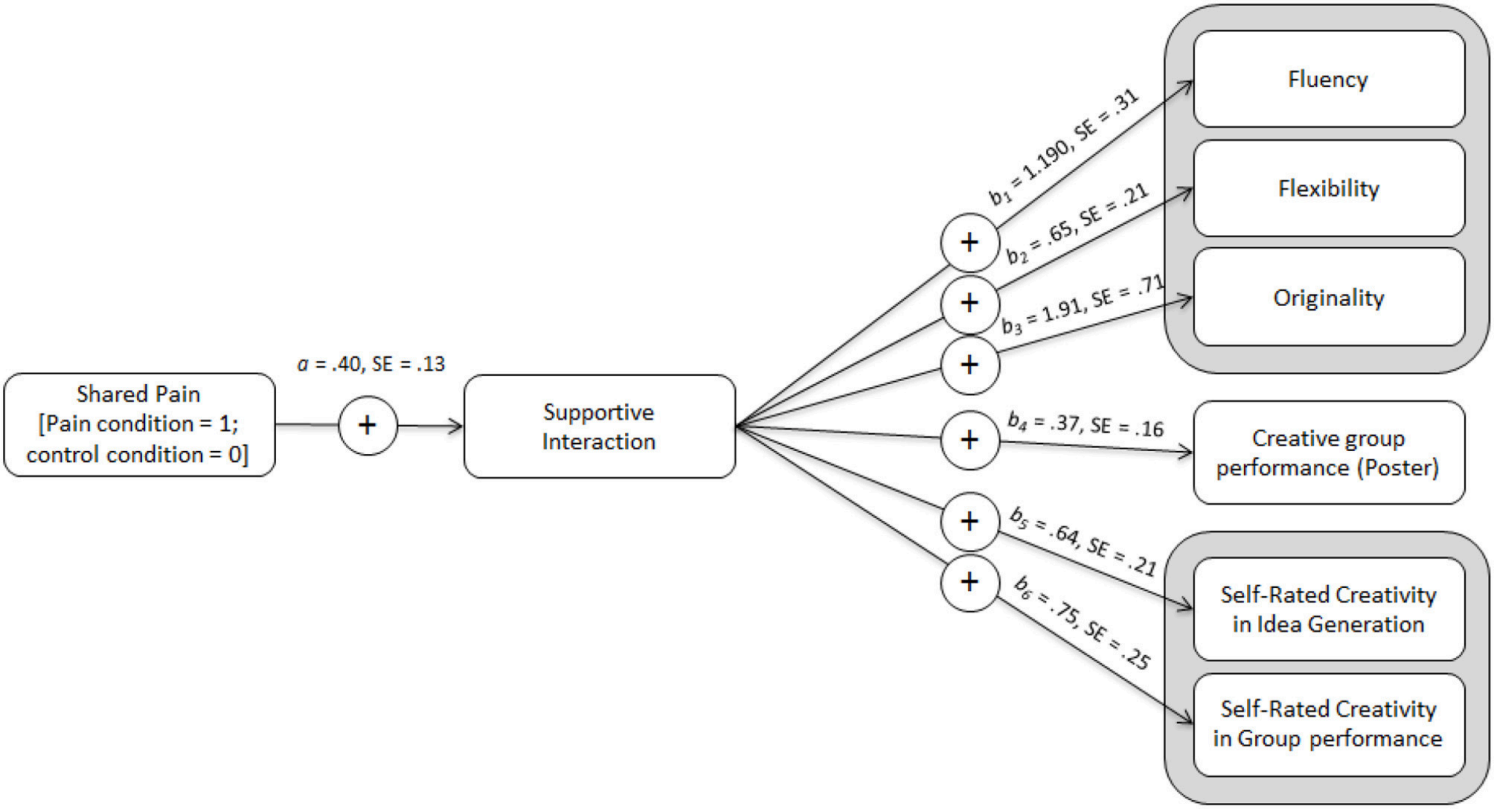
Mediation displaying coefficients for paths from shared pain through supportive interaction to (a) creative idea generation (fluency, flexibility, and originality), (b) creative group performance in poster generation task, and (c) self-perceived creativity (in idea generation and group performance).

In line with our predictions we examined the indirect effect of pain (vs. no-pain) on each index of creativity through increased supportive interaction within the team. Focusing first on the measures of creativity in the brick task, mediation analyses based on 5,000 bootstrapped samples using bias-corrected and accelerated 95% confidence intervals (Preacher and Hayes, 2008) showed that condition (direct effects in brackets) had a significant indirect effect (via supportive interaction) on fluency, *IE* = 0.485, 95% CI[0.178,0.992] (cond*DE* = −0.066, 95% CI[−0.696,0.564]), flexibility, *IE* = 0.262, 95% CI[0.081,0.606] (cond*DE* = 0.048, 95% CI[−0.389,0.485]), and originality, *IE* = 0.776, 95% CI[0.221, 1.945] (cond*DE* = −0.012, 95% CI[−1.471, 1.448]). Using this same model, we examined any indirect effect of condition on the ratings of poster creativity. This also revealed a significant indirect effect, *IE* = 0.151, 95% CI[0.024,0.347] (cond*DE* = 0.015, 95% CI[−0.316,0.344]). Finally, we examined whether condition predicted participants' own perceptions of creativity in each task. This again revealed a significant indirect effect of condition through supportive interaction on perceptions of creativity in the idea brainstorming task, *IE* = 0.259, 95% CI[0.072,0.623] (cond*DE* = 0.039, 95% CI[−0.396,0.475]), and the poster task, *IE* = 0.306, 95% CI[0.091,0.715] (cond*DE* = −0.161, 95% CI[−0.670,0.350]). These effects remained significant when controlling for team size (see Figure 1).

Consistent with the lack of any direct effects of condition on creativity, examination of experimental group differences using *t*-tests revealed no effect of pain on any measure of creativity. For the brick task there was no difference across conditions in fluency [pain: *M* = 4.15, *SD* = 1.24; control: *M* = 3.73, *SD* = 1.17, *t*_(55)_ = 1.29, *p* = 0.203], flexibility [pain: *M* = 3.15, *SD* = 0.82; control: *M* = 2.84, *SD* = 0.78, *t*_(55)_ = 1.44, *p* = 0.155], or originality [pain: *M* = 6.81, *SD* = 2.80; control: *M* = 6.05, *SD* = 2.41, *t*_(55)_ = 1.08, *p* = 0.284]. Examination of the independent poster ratings revealed no differences across conditions in third party rated creativity [pain: *M* = 3.69, *SD* = 0.65; control: *M* = 3.52, *SD* = 0.50, *t*_(55)_ = 1.06, *p* = 0.295], blandness [pain: *M* = 3.61, *SD* = 0.53; control: *M* = 3.79, *SD* = 0.56, *t*_(55)_ = 1.25, *p* = 0.217], or richness [pain: *M* = 3.57, *SD* = 0.54; control: *M* = 3.50, *SD* = 0.47, *t*_(55)_ = 0.51, *p* = 0.613]. Finally, examination of participants' own ratings of creativity revealed no significant differences across conditions in perceptions of creativity in the brainstorming task [pain: *M* = 5.08, *SD* = 0.76; control: *M* = 4.78, *SD* = 0.83, *t*_(55)_ = 1.40, *p* = 0.169] or in the poster task [pain: *M* = 5.33, *SD* = 0.86; control: *M* = 5.19, *SD* = 1.00, *t*_(55)_ = 0.58, *p* = 0.563].

## Discussion

The findings of our experiment demonstrate that sharing aversive experiences of pain can enhance supportive team interaction and that this in turn promotes creativity. We find this effect across multiple converging indices of creativity and across two different tasks drawing on very different creative outcomes. Moreover, our indices come from a range of different sources including structured and objective ratings of creativity, third party ratings of creative content, and subjective ratings of the team's output. Also noteworthy is that this (indirect) effect of shared pain through supportive interaction to creativity arises when compared to teams that shared very similar but non-painful experiences. Together the converging nature of these results provides support for our predictions.

Our findings corroborate past research showing that shared pain increases trust and cooperation within groups (Bastian et al., 2014a). We extend this previous work by examining how individuals interact within these groups, finding that they engage in more supportive social interaction (indicated by ratings of eye-contact, talking, comfort, cohesion, helping, encouragement, contribution, cooperation). In short, this shows that beyond building trust and cooperation within groups, shared pain can also build supportive team climates. Furthermore, we show that this has the downstream effect of increasing team creativity.

Our findings shed new light on past work examining increases in trust and cooperation within groups that share painful experiences. Responding to shared pain by bonding to one's group is likely a functional response to threatening environments (e.g., Turner et al., 1984; Taylor et al., 2000; Williams, 2007) facilitating the emergence of pro-group behavior (Whitehouse et al., 2014, 2017). This provides a possible explanation for why painful social rituals persist within a range of cultural contexts (Durkheim, 1912/1995). Yet, our findings suggest that beyond simply strengthening groups to become resilient against threat, sharing a painful experience also fosters a supportive environment within those groups. People are not only more likely to act in ways that protect their own interests and the interests of their group members, they are also motivated to affiliate and to build supportive relationships. This in turn means that group members become more attuned and attentive to each other fostering an environment characterized by mutual support. It is tempting to consider that responding to threats in a way that builds the capacity for creativity and innovation represents a functional response: it bolsters group-based competencies and survival advantages that may be effective in responding to challenging environments.

It is noteworthy that we did not find a main effect of shared pain (vs. shared similar experiences with no pain) on creativity. Rather, effects of pain occurred via an associated increase in supportive social interaction. There are few reasons to expect that pain alone should increase group creativity (save for a functionalist account as detailed above), but rather that creativity may be one type of group competency which results from the supportive group environment which arises in response to shared pain. Nonetheless, although non-significant, mean scores for all indices of creativity were higher in the pain condition, indicating that pain may influence creativity, but that this is best explained via its role in building supportive social environments within groups.

Our findings provide for some reflection on current approaches to team-building within group and organizational settings. There is a current trend toward making office environments fun and comfortable, and team building exercises revolve around sharing in positive rewards. There are clearly benefits to these approaches, but our work suggests that aversive experiences may be an important, yet frequently overlooked, avenue for achieving similar ends. There are two reasons to take note of our findings in this regard. First, although we did not directly test the effects of shared positive vs. shared negative experiences, our primary intent was to examine whether negative experiences, which are often dismissed as detracting from positive outcomes, have the capacity to build the kind of supportive team environments that support the creative process. This provides a different perspective from which to think about and respond to such experiences. Second, teams are frequently exposed to shared negative experiences, such as adverse task demands or negative feedback. Indeed, failure is a common, and some might say necessary, feature of innovative environments. Understanding that these set-backs may also feed into creating team environments that are more conducive to future success provides a different and perhaps more helpful perspective to work from. We suggest that research on team creativity might begin to examine group processes associated with creativity in similar ways that researchers have begun to think about processes associated with resilience—as an effective response to challenging and sometimes adverse experiences (Kalisch et al., 2017).

We see several avenues for future research to build upon these initial observations. First, we used a particular type of aversive experience—physical pain. While this presents a prototypical aversive experience and is therefore ideal for an initial investigation of the concept, it would be good to examine the extent to which similar findings are obtained for other types of aversive experiences. Although teams may be exposed to physical pain in the context of team building activities (e.g., boot camps) or when their primary activity is physical in nature (e.g., army training), many teams are more likely to experience adversity through cognitively challenging tasks, tight timelines, heavy workloads, or perhaps even difficult bosses. Whether these other forms of adversity have similar effects would be important to know.

Second, it would be important to directly compare the effects of adverse experiences with more pleasant experiences. As we note at the beginning, there is good theoretical reason to expect that sharing an adverse experience may be especially likely to foster supportive team social interaction. This is because in the context of adverse events, people are motivated to seek affiliation with, and provide support to, others within their social environment. This in turn elicits interdependency between group members and foments group commitment. Nonetheless, as we note, there is some evidence that positive experiences may have similarly beneficial consequences. One possibility is that it is the intensity of the experience, rather than its positivity or negativity which explains these effects. Yet, as we detail above, the theoretical case for why adverse experiences in particular should foster supportive interactions is stronger than for the positivity or intensity of an experience. In any case, it would be important to examine the degree to which various shared positive and negative experiences have the capacity to produce different forms of supportive interactions, and in turn foster creative processes in teams. As we note above, teams are frequently exposed to adverse circumstances and knowing that these events hold the potential for positive team outcomes (in addition to positive experiences) is informative and advantageous.

Third, we have characterized our findings as focused on a shared experience of adversity. We see this as the best way to describe the nature of the experience, which involved conjointly undertaking two different tasks in a group context. We note, however, that this manipulation did not involve interdependency in the management of adverse outcomes (e.g., such as may be the case when one person's actions can cause adverse outcomes for another). Although we do not have a strong reason to expect that there would be differential effects of these two types of shared experience (interdependent vs. group context) future research could examine potential differences in the observed effects using these different approaches.

Finally, in the present research, we examined core indicators of creativity in the form of ideational fluency (in the brainstorming task) and novel task-based creativity (in the poster creation task). Beyond these, in future work there would be value in examining how aversive experiences and associated team supportive environment are related to a range of other forms of creativity such as the creation of useful new ideas or products (Amabile, 1996; Runco, 2004), insight in solving complex problems (De Dreu et al., 2008) and little (everyday) and big (eminent) C creativity (Beghetto and Kaufman, 2007).

Overall, our findings provide novel insight into the ways in which adverse experiences shape group processes. Beyond leading people to bond together, cooperate, and increase their pro-group behavior, sharing an averse painful experience with others can also shape group processes—increasing supportive team environments—and thereby promoting enhanced creativity within groups.

## Author contributions

BB, JJ, and NS conceptualized and designed the study. HT collected the data. BB and NS analyzed the data. BB, NS, and JJ wrote the manuscript.

### Conflict of interest statement

The authors declare that the research was conducted in the absence of any commercial or financial relationships that could be construed as a potential conflict of interest.

## Footnotes

^1^Given that our sample included twice as many females than males, we also explored whether gender might have influences our main findings. Across all analyses entering gender as a co-variate did not change any of the reported results and gender was largely unrelated to our dependent variables.

^2^Across all analyses entering group size as a covariate did not change any of the reported results. Group size was size was unrelated to all dependent variables, except for ratings of poster creativity (*r* = 27*, p* = 0.044), but again, including it as a covariate did not impact on the report findings.

^3^Participants also rated their perception of group achievement on 4-items for both the brick and the poster tasks. As these were not related to our focus on creativity we did not analyze these data further.

^4^At the end of the study participants also rated their bonding on the same seven statements used in Bastian et al. (2014a). Analysis revealed that there were no differences across conditions on this measure (pain: *M* = 4.95, *SD* = 0.91; no-pain: *M* = 4.90, *SD* = 1.03; *t* = 0.35, *p* = 0.730). Given that these self-ratings occurred after the two lengthy creativity tasks, we felt that responses on this measure would have been primarily determined by interactions within the creativity tasks, rather than the experimental effects of pain vs. no-pain.

^5^This measure also includes a subscale related to loss (*depressing, pitiful, painful, intolerable*) which seemed less conceptually related to our approach to capturing an aversive experience. Furthermore, the items painful and intolerable would have captured pain responses rather than loss in our study, leading to a lack of conceptual clarify.

^6^Participants were also asked to rate how they felt at the end of the study, however we did not analyse these data as we were primarily interested in their experience of the experimental tasks.
