# Supplementary material for: Shared Adversity Increases Team Creativity Through Fostering Supportive Interaction
Source: Front Psychol. 2018 Nov 23;9:2309. doi: 10.3389/fpsyg.2018.02309 (PMC6266683; doi:10.3389/fpsyg.2018.02309)
Supplement: Supplementary file 1 [file Data_Sheet_1.pdf]

Supplementary Materials

Coding Manual for Coding of Videos:

Coding scale: 1 = Not at all, 3 = neutral, to 5 = A lot.

- Eye Contact (1- Not a lot of eye contact, 5 – A lot of eye contact): the amount of eye contact (include gazing at others' faces) a participant made.

- Inhibition, Comfortable, Guarded: coders assess participants' behaviors, facial expression, tones of voice, etc. (For Post Task – part 2, where participants performed the task in silence, default rating of inhibition = 1, guarded = 1 and comfortable = 3 were given).

    - Inhibition (1 – Not at all inhibited, 5 – Very inhibited): inhibited participants are timid and uncomfortably quiet.
    - Comfortable (1- Not at all comfortable, 5 – Very comfortable): participant would be more open, talk more, laugh more easily and don't appear guarded or inhibited.
    - Guarded (1- Not at all guarded, 5 – Very guarded): Guarded participants would be uncomfortable quiet and also put on a defensive front.

- Encourage (1 – Not at all encouraging, 5 – very encouraging): Nodding, verbal agreement, verbal elaboration, appreciative smiling, laughing was counted as gestures of encouragement when they are made in response to someone's behavior/comments.

- Contribution (1- No Contribution at all, 5 – A lot of contribution): includes verbal comments, elaboration. How much effort does a person seem to put towards a task. In the poster tasks, we also count whether someone is willing to move around, do different tasks while working on the poster. For Poster Task – part 2, most people will get a default score of 3, unless it looks like they do considerably less work than their team members.

- Talk (1 – not at all, 5 – a lot): Small talk that does not include task-related communication

- Help (1 – no help at all, 5 – a lot of help): prosocial helping behavior (passing others stuff, pour water for others, help clarifying someone's ideas, etc.)

- Group Cohesion (1 – No cohesion at all, 5 – a lot of cohesion): a cohesive group would be responsive to one another's contribution. There would be a lot of eye contact, verbal and non-verbal agreement, and encouraging behaviors. For Poster Task – part 2, most groups will get default rating of 3.

- Group Cooperation (1 – No cooperation at all, 5 – a lot of cooperation): in Poster – part 2, most groups will get 3 for this variable. Though some groups might have more complex/creative work distribution system that requires more coordination and pre-planning and they will get a higher score.

- Group Enjoyment (1 – No enjoyment at all, 5 – a lot of enjoyment): participants' facial expressions, verbal and non-verbal communication during the tasks, how often they smile, laugh. In Poster task – part 2, most groups will get 3 for this variable.
